# Supplementary material for: Reappraisal of sauropod dinosaur diversity in the Upper Cretaceous Winton Formation of Queensland, Australia, through 3D digitisation and description of new specimens
Source: PeerJ. 2024 Apr 9;12:e17180. doi: 10.7717/peerj.17180 (PMC11011616; doi:10.7717/peerj.17180)
Supplement: Supplemental Information 1 [file peerj-12-17180-s001.docx]

# **Reappraisal of sauropod dinosaur diversity in the Upper Cretaceous Winton Formation of Queensland, Australia, through 3D digitisation and description of new specimens**

Samantha L. Beeston^1,2,3^, Stephen F. Poropat^4^, Philip D. Mannion^1^, Adele H. Pentland^3,4^, Mackenzie J. Enchelmaier^3^, Trish Sloan^3^, David A. Elliott^3^

^1^Department of Earth Sciences, University College London, London, United Kingdom

^2^Faculty of Science, Engineering and Technology, Swinburne University of Technology, Hawthorn, Victoria, Australia

^3^Australian Age of Dinosaurs Museum of Natural History, Winton, Queensland, Australia

^4^Western Australian Organic and Isotope Geochemistry Centre, School of Earth and Planetary Science, Curtin University, Bentley, Western Australia, Australia

Corresponding author:

Samantha L. Beeston

# E-mail: samanthalbeeston@gmail.com

# **TABLE S1. Winton Formation sauropod body fossils collected by the AAOD 1999–2022**.

Some material remains unprepared and is not described herein.

| **Specimen and Locality** | **Locality in Queensland and year(s) collected** | **Material** |
| --- | --- | --- |
| AODF 0004 | AODL 0004, Belmont Station, 1998. | Left metacarpal III. |
| AODF 2851 | QM L1333/ AODL 0001, Belmont Station, Winton; southern part of the ‘Elliot’ site, a.k.a. ‘Elliot site’ proper, 1999–2004. | Caudal vertebra. |
| AODF 2854 | QM L1333/ AODL 0001, Belmont Station, Winton; southern part of the ‘Elliot’ site, a.k.a. ‘Elliot site’ proper, 1999–2004. | Right metacarpal IV. |
| QM F43302 ‘Elliot’ | QM L1333/ AODL 0001, Southern part of the ‘Elliot’ site, Belmont Station, Winton; a.k.a. ‘Elliot site’ proper, 1999–2004. | Right femur. |
| QM F44573 | QM L1333/ AODL 0001, Southern part of the ‘Elliot’ site, Belmont Station, Winton; a.k.a. ‘Elliot site’ proper, 1999–2004. | Left tibia. |
| Multiple specimens | AODL 0126, Middle section of the ‘Elliot’ site, Belmont Station, Winton; a.k.a. ‘Kylie’s Corner”, 1999–2004. | Cervical vertebra; dorsal vertebra; and radius. |
| AODF 836 ‘Alex’ *Diamantinasaurus* referred specimen (tentatively includes a tooth catalogued as AODF 2298) | AODL 0127, Northern part of the ‘Elliot’ site, Belmont Station, Winton, 1999–2004. | Left squamosal; right and left quadrates; tooth (AODF 2298); braincase [including left frontal, both parietals, supraoccipital, otoccipitals (exoccipitalopisthotics), basioccipital, partial basisphenoid, prootics, laterosphenoids and orbitosphenoids]; right surangular; atlas intercentrum; axis; cervical vertebrae III–VI (IV and V fragmentary); middle cervical neural arch; four dorsal vertebrae; dorsal ribs; two co-ossified sacral centra; partial right scapula; right and left iliac preacetabular processes; right and left pubes; right and left ischia; and abundant associated fragments, many constituting partial ribs or vertebrae. |
| AODF 0009 | AODL 0009, Belmont Station, Winton, 2001. | ?right ulna. |
| AODF 0032 ‘Mick’ | AODL 0049, ‘Mick’ site, unidentified sheep station, Winton, 2003. | Three incomplete cervical vertebrae; eight incomplete caudal vertebrae; left humerus, left pubis; left ischium; and associated fragments. |
| AODF 660 ‘Wade’ *Savannasaurus* holotype | AODL 0082, ‘Ho-Hum’ site, Belmont Station, Winton 2005, 2012. | One posterior cervical vertebra; several cervical ribs; dorsal vertebrae III–X; several fragmentary dorsal ribs; at least four coalesced sacral vertebrae with processes; at least five partial caudal vertebrae; fragmentary scapula; left coracoid; left and right sternal plates; incomplete left and right humeri; fragmentary ulna; left radius; left metacarpals I–V; right metacarpal IV; two manual phalanges; iliac fragments; co-ossified left and right pubes and ischia; left astragalus; right metatarsal III; associated fragments. |
| AODF 661 ‘Packer’ | AODL 0083, ‘Boxing Day’ site, Belmont Station, Winton, 2005. | Humerus; and partial limb elements. |
| AODF 591 ‘Bob’ | AODL 0080, ‘Bob’ site, Belmont Station, Winton, 2006. | Caudal vertebrae; partial scapula; two dorsal ribs; unidentified girdle element; metapodial; and partial left fibula. |
| AODF 590 ‘McKenzie’ | AODL 0079, ‘McKenzie’ site, Elderslie Station, Winton, 2006. | Fragmentary caudal vertebra; femur distal condyles; right tibia; right fibula; proximal and distal left tibia and fibula; and surface fragments. |
| AODF 603 ‘Matilda’ *Diamantinasaurus* holotype | AODL 0085, ‘Matilda’ site, Elderslie Station, Winton, 2006–2010. | Dentary fragment; tooth; three partial cervical ribs; three incomplete dorsal vertebrae; dorsal ribs; fragmentary gastralia; five coalesced sacral vertebrae; isolated sacral processes; left and right scapulae; right coracoid; sternal plate; left and right humeri; left and right ulnae; right radius; left and right metacarpals I–V; eight manual phalanges (including right manual ungual I-2); left ilium; left and right pubes; left and right ischia; right femur; right tibia; right fibula; and right astragalus. |
| AODF 656 ‘Dixie’ | AODL 0117, ‘Dixie’ site, Elderslie Station, Winton, 2011. | Axial and appendicular elements including cervical, dorsal and sacral vertebrae; partial left scapula; and right ulna. |
| AODF 657 ‘Munro’ | AODL 0118, ‘Munro’ site, Elderslie Station, Winton, 2010. | Metatarsal; and surface fragments. |
| AODF 663 ‘Oliver’ *Diamantinasaurus* referred specimen | AODL 0122, ‘Oliver’ site, Elderslie Station, Winton, 2012. | Left cervical rib; three dorsal vertebrae; dorsal ribs; left scapula; right humerus; right manual ungual phalanx; and right femur. |
| AODF 662 ‘Olga’ | AODL 0121, ‘Olga’ site, Elderslie Station, Winton, 2012. | Vertebral fragments; and incomplete ulna. |
| **Specimen and Locality** | **Locality in Queensland and year(s) collected** | **Material** |
| AODF 665 ‘Trixie’ | AODL 0125, ‘Pete’ site, Elderslie Station, Winton, 2012, 2013. | Axial and appendicular elements including dorsal ribs; right ulna; phalanx; paired pubes; right femur; right tibia; and right fibula. |
| AODF 2306 | AODL 0317, Elderslie Station, 2013. | Caudal vertebra. |
| AODF 832 ‘Patrice’ | AODL 0160, ‘Patrice’ site, Lovelle Downs Station, Winton, 2014. | Cervical rib; caudal vertebra; right femur; and additional bones in concretion. |
| AODF 844 ‘Ian’ | AODL 0215, ‘Ian’ site, Elderslie Station, Winton, 2015. | Right scapula; and right coracoid. |
| AODF 845 ‘Kate’ | AODL 0216 ‘Kate’ site, Elderslie Station, Winton, 2015. | Dorsal rib; partial fibula; fragmented metatarsals; and associated fragments. |
| AODF 848 ‘Matt’ | AODL 0219, ‘Matt’ site, Elderslie Station, Winton, 2015. | Vertebrae; ribs; and limb bones in concretion. |
| AODF 666 ‘Devil Dave’ | AODL 0128 ‘Devil Dave’ site, Belmont Station, Winton, 2016–2017. | Right tibia; fibula fragments; right astragalus; and surface fragments. |
| AODF 888 ‘Judy’ | AODL 0246 ‘Son of the Devil (S.O.D.)’ site, Belmont Station, 2017. | Indeterminate skull fragment; six teeth; cervical vertebrae III–XV; dorsal vertebrae I–X; dorsal ribs; sacrum; left and right scapulae, coracoids and sternal plates; left humerus; right ulna; left metacarpal II; manual non-ungual phalanx; left ilium; left pubis; ?ischium; left femur; left tibia; left fibula; left astragalus; additional fragments; integument; and gut contents. |
| AODF 2296 ‘Leo’ | AODL 0247 ‘Leo site’, Belmont Station, Winton, 2017, 2021–2022. | 20 caudal vertebrae; five chevrons; dorsal ribs; left coracoid; left ulna; right radius; left metacarpal IV; proximal right fibula; and associated fragments. |
| AODF 906 ‘Ann’ *Diamantinasaurus* referred specimen | AODL 0252 ‘Ann’ site, Elderslie Station, Winton, 2018. | Left premaxilla; left maxilla; left lacrimal; left and right postorbitals; left and right squamosals; left and right quadratojugals; left and right quadrates; left and right pterygoids; left ectopterygoid; braincase (including left frontal, left parietal, supraoccipital, partial exoccipital–opisthotics, fragmentary basioccipital, prootics, laterosphenoids, and orbitosphenoids); left and right dentaries; left surangular; dorsal ribs; one chevron; both femora; both tibiae; both fibulae; a probable right astragalus; right metatarsals I and III–V; right pedal phalanges I-1, I-2 (ungual), III-1, IV-1, and IV-2; and associated fragments. |
| AODF 960 ‘Mitchell’ | AODL 0270, ‘Mitchell’ site, Elderslie Station, Winton, 2019, 2021–2022. | At least 22 teeth; and associated postcranial axial and appendicular elements. |

**Table S2. Scapulae measurements**

**of AODF 0603, AODF 0656 and AODF 0844.** An asterisk (*) indicates a measurement taken from an incomplete element.

| Measurements (mm) | AODF 0603 left scapula | AODF 0603 right scapula | AODF 0656 left scapula | AODF 0844 right scapula |
| --- | --- | --- | --- | --- |
| Proximodistal length | 1100* | >1280 | - | 930 |
| Dorsoventral height of blade distal end | 301* | 330 | - | 252 |
| Mediolateral thickness of blade distal end | 41* | 34 | - | 36 |
| Glenoid fossa dorsoventral height | - | 183 | 216* | 212 |
| Coracoid articulation dorsoventral height | - | 225 | 269 | 338 |
| Distance from ventral process to base of glenoid | - | 182 | 205 | 188 |

**Table S3. Coracoid measurements**

**of AODF 0603 (adapted from Poropat et al. [2015b]), AODF 0844 and AODF 2296.** An asterisk (*) indicates a measurement taken from an incomplete element.

| Measurements (mm) |  | AODF 0603 right coracoid | AODF 0844 right coracoid | AODF 2296 left coracoid |
| --- | --- | --- | --- | --- |
| Maximum length | Proximodistal | 430 | 298* | 262* |
|  | Dorsoventral | 340 | 462* | 423 |
| Contribution to glenoid | Dorsoventral | 198 | 130 | 125 |
| Scapular articulation | Dorsoventral | 220 | 332 | 180 |
| Coracoid foramen diameter | Proximodistal | 60 | 56 | 55 |
|  | Dorsoventral | 60 | 34 | 28 |
| Coracoid foramen thickness at posterior margin |  | 30 | 42 | 32 |
| Distance between coracoid foramen ventral margin and glenoid fossa posterior margin |  | 145 | 118* | 159 |

**Table S4. Ulnae measurements**

**of AODF 0603 (adapted from Poropat et al. [2015b]), AODF 0656, AODF 0665 and AODF 2296.** An asterisk (*) indicates a measurement taken from an incomplete element.

| Measurements (mm) | AODF 0603 left ulna | AODF 0603 right ulna | AODF 0656 right ulna | AODF 0665 right ulna | AODF 2296 left ulna |
| --- | --- | --- | --- | --- | --- |
| Proximodistal length | 809 | 700 | 883 | 599* | 273* |
| Proximal end maximum mediolateral width | 350 | 305 | 330 | 267* | 91* |
| Proximal end maximum anteroposterior width | 288 | 305 | 299 | 210* | 129* |
| Distal end maximum mediolateral width | 192 | 172 | 183 | 175* | 111* |
| Distal end maximum anteroposterior width | 165 | 170 | 143 | 114* | 80* |

**Table S5. Metacarpal measurements**

**of AODF 0603 (adapted from Poropat et al. [2015b]), AODF 2854 and AODF 2296.**

| Measurements (mm) | | AODF 0603 |  |  |  |  |  |  |  |  |  | AODF 2854 | AODF 2296 |
| --- | --- | --- | --- | --- | --- | --- | --- | --- | --- | --- | --- | --- | --- |
|  |  | Left I | Left II | Left III | Left IV | Left V | Right I | Right II | Right III | Right IV | Right V | Right IV | Left IV |
| Proximodistal length |  | 372 | 375 | 412 | 352 | 325 | 347 | 355 | 388 | 349 | 343 | 273 | 259 |
| Proximal end mediolateral width | | 174 | 109 | 150 | 102 | 133 | 175 | 121 | 151 | 112 | 171 | 85 | 88 |
| Proximal end anteroposterior length | | 128 | 151 | 154 | 157 | 93 | 111 | 161 | 151 | 161 | 122 | 106 | 98 |
| Mid-shaft mediolateral width | | 104* | 88 | 83 | 79 | 75 | 88 | 89 | 80 | 86 | 88 | 62 | 65 |
| Mid-shaft anteroposterior length | | 62* | 66 | 92 | 69 | 58 | 60 | 50 | 71 | 66 | 57 | 55 | 42 |
| Distal end mediolateral width | | 141 | 153 | 173 | 167 | 121 | 132 | 141 | 152 | 152 | 124 | 122 | 81 |
| Distal end anteroposterior length | | 115 | 104 | 114 | 97 | 100 | 113 | 95 | 81 | 96 | 85 | 55 | 44 |

**Table S6. Right manual phalange measurements**

**of AODF 0603.**

| Measurements (mm) | Right II-1 | Right III-1 | Right IV-1 |
| --- | --- | --- | --- |
| Maximum proximodistal length | 70 | 80 | 62 |
| Maximum mediolateral width | 114 | 100 | 91 |
| Maximum anteroposterior length | 58 | 60 | 63 |
| Anterior mediolateral width | 46 | 56 | 61 |
| Anterior maximum proximodistal length | 31 | 27 | 24 |
| Posterior mediolateral width | 114 | 100 | 91 |
| Posterior maximum proximodistal length | 70 | 80 | 59 |

**Table S7. Caudal vertebrae measurements**

**of AODF 2296.** An asterisk (*) indicates a measurement taken from an incomplete element.

| Measurements (mm) | |  | A | B | C | D | E | F | G | H | I | J | K | L | M | N | O | P | Q | R | S | T |
| --- | --- | --- | --- | --- | --- | --- | --- | --- | --- | --- | --- | --- | --- | --- | --- | --- | --- | --- | --- | --- | --- | --- |
| Centrum | Anteroposterior length | | 81* | 95 | 101 | 95 | 82* | 85* | 95 | 100 | 100 | 103 | 96 | 106 | 98 | 97 | 99 | 85 | 73 | 71 | 63* | 21* |
|  | Anterior | Dorsoventral height | 146 | 126 | 127 | 99 | 103 | 115 | 90 | 98 | 85 | 86* | 78 | 77 | 73 | 71 | 67 | 46 | 36 | 34 | 24* | - |
|  |  | Transverse width | 161 | 157 | 130 | 122 | 125 | 111 | 98 | 102 | 91 | 92 | 84 | 80 | 75 | 74 | 69 | 50 | 39 | 28* | 29* | - |
|  |  | Height: width ratio | 0.91 | 0.80 | 0.98 | 0.81 | 0.82 | 1.04 | 0.92 | 0.96 | 0.93 | 0.93* | 0.93 | 0.96 | 0.97 | 0.96 | 0.97 | 0.92 | 0.92 | 1.21* | 0.83* | - |
|  | Posterior | Dorsoventral height | 129* | 134 | 120 | 92 | 92* | 95* | 86 | 74* | 79 | 93 | 68* | 74 | 67 | 66 | 64 | 42 | 26* | 30 | 18* | 33 |
|  |  | Transverse width | - | 141 | 129 | 112 | 114* | 93* | 89 | 99 | 83 | 85 | 88* | 75 | 71 | 72 | 70 | 40* | 28* | 27* | 26* | 34 |
|  |  | Height: width ratio | - | 0.95 | 0.93 | 0.82 | 0.80* | 1.02 | 0.97 | 0.75* | 0.95 | 0.98 | 0.77* | 0.99 | 0.94 | 0.92 | 0.91 | 1.05* | 0.93* | 1.11* | 0.69* | 0.97 |
| Neural canal | Anterior | Dorsoventral height | 19* | 50 | 46 | - | - | 46 | - | 25* | - | - | - | 25 | - | 21 | 13* | 16 | - | - | - | - |
|  |  | Transverse width | 45* | 43 | 39 | 26* | - | 33 | - | 25 | 22* | - | 22* | 23 | 19 | 20 | 18 | 14 | 17* | 15* | - | - |
|  | Posterior | Dorsoventral height | - | 47 | 40 | - | - | 40 | - | - | - | - | - | 27 | - | 22 | - | 18 | - | - | - | - |
|  |  | Transverse width | - | 36 | 29 | 28* | - | 32 | - | 24 | 17* | - | - | 27 | 18 | 25 | 17 | 15 | 16* | 17 | - | - |
| Neural spine | Dorsoventral height | | - | 42 | - | - | - | 26 | - | - | - | - | - | 18 | - | 99 | - | 13 | - | - | - | - |
|  | Anteroposterior length | | - | 36 | - | - | - | 30 | - | - | - | - | - | 59 | - | 67 | - | 50 | - | - | - | - |
|  | Transverse width | | - | 20 | - | - | - | 8 | - | - | - | - | - | 12 | - | 69 | - | 15 | - | - | - | - |

**Table S8. Sternal plate measurements**

**of AODF 2296.** An asterisk (*) indicates a measurement taken from an incomplete element.

| Measurements (mm) | Left sternal plate |
| --- | --- |
| Maximum mediolateral length | 386* |
| Maximum dorsoventral length | 548* |
| Maximum proximal thickness | 17 |
| Maximum distal thickness | 9 |

**Table S9. Radius measurements**

**of AODF 2296**. An asterisk (*) indicates a measurement taken from an incomplete element.

| Measurements (mm) | Right radius |
| --- | --- |
| Length | 518* |
| Maximum proximal mediolateral breadth | 103* |
| Maximum proximal anterior-posterior length | 101* |
| Mid-shaft mediolateral breadth | 81 |
| Mid-shaft anterior-posterior length | 53 |
| Maximum distal mediolateral breadth | 136* |
| Maximum distal anterior-posterior breadth | 87* |

**Table S10. Fibulae measurements**

**of AODF 0590, AODF 0591, AODF 0665 and AODF 2296.** An asterisk (*) indicates a measurement taken from an incomplete element.

| Measurements (mm) | AODF 0590 right fibula | AODF 0591 left fibula | AODF 0665 right fibula | AODF 2296 right fibula |
| --- | --- | --- | --- | --- |
| Proximodistal length | 989 | - | 888 | - |
| Maximum proximal mediolateral width | 132 | 85 | 82 | 49* |
| Maximum proximal anteroposterior breadth | 261 | 144* | 245 | 127* |
| Mid-shaft mediolateral width | 80 | 55 | 48* | - |
| Maximum distal mediolateral width | 148 | - | 142 | - |
| Maximum distal anteroposterior breadth | 183 | - | 214* | - |
| Robusticity | 0.12 | - | 0.13* | - |

**Table S11. Tibiae measurements**

**of AODF 0590, AODF 0665 and AODF 0666.** An asterisk (*) indicates a measurement taken from an incomplete element.

| Measurements (mm) | AODF 0590 right tibia | AODF 0665 right tibia | AODF 0666 right tibia |
| --- | --- | --- | --- |
| Proximodistal length | 1040 | 893* | 917* |
| Maximum proximal mediolateral width | 275 | 323* | 162 |
| Maximum proximal anteroposterior breadth | 272 | 259* | 247 |
| Mid-shaft mediolateral width | 173 | 179 | 128* |
| Maximum distal mediolateral width | 376 | 374 | 220 |
| Maximum distal anteroposterior breadth | 195 | 172 | 126 |
| Robusticity | 0.26 | 0.33* | 0.19* |

**Table S12. Caudal vertebrae measurements**

**of AODF 0032, AODF 0590, AODF 0591, AODF 0832, AODF 2306 and AODF 2851**. An asterisk (*) indicates a measurement taken from an incomplete element.

| Measurements (mm) | |  | AODF 0032 |  |  |  |  |  |  |  | AODF 0590 | AODF 0591 |  | AODF 0832 | AODF 2306 | AODF  2851 |
| --- | --- | --- | --- | --- | --- | --- | --- | --- | --- | --- | --- | --- | --- | --- | --- | --- |
|  |  |  | A | B | C | D | E | F | G | H |  | A | B |  |  |  |
| Centrum | Anteroposterior length | | 124 | >110 | >78 | 118 | 100 | 158 | 141 | 131 | 170 | 124 | >110 | 95 | 120 | 94 |
|  | Anterior | Dorsoventral height | 212 | 187 | - | - | 120 | - | - | - | 221 | 212 | 187 | - | 89 | 64 |
|  |  | Transverse width | 212 | 215 | - | - | 160 | 115 | 105 | 110 | - | 212 | 215 | 115 | 68 | 58 |
|  |  | Height: width ratio | 1 | 0.87 | - | - | 0.75 | - | - | - | - | 1 | 0.87 | - | 1.30 | 1.1 |
|  | Posterior | Dorsoventral height | 206 | 174 | >153 | 145 | - | - | - | - | - | 206 | 174 | 116* | 88 | 53 |
|  |  | Transverse width | 242 | 200 | 193 | 171 | - | 110 | - | >82 | - | 242 | 200 | 126 | 73 | 59 |
|  |  | Height: width ratio | 0.85 | 0.84 | >0.79 | 0.85 | - | - | - | - | - | 0.85 | 0.84 | 0.92* | 1.21 | 0.90 |
| Neural canal | Anterior | Dorsoventral height | - | ~60 | - | 46 | - | - | - | - | - | - | - | - | 16 | - |
|  |  | Transverse width | 40 | 42 | - | 36 | - | - | - | - | - | - | - | 26 | >20 | 15 |
|  | Posterior | Dorsoventral height | - | 52 | - | 40 | - | - | - | - | - | - | - | - | 28 | - |
|  |  | Transverse width | 42 | 37 | - | 45 | - | - | - | - | - | - | - | 25 | 13 | 8 |
| Neural spine | Dorsoventral height | | - | >101 | - | - | - | - | - | - | - | - | - | 28 | - | - |
|  | Anteroposterior length | | - | >129 | - | - | - | - | - | - | - | - | - | 60 | - | - |
|  | Transverse width | | - | 54 | - | - | - | - | - | - | - | - | - | 14 | - | - |

**Table S13. Pubis and ischium measurements**

**of AODF 0032 and AODF 0665.** An asterisk (*) indicates a measurement taken from an incomplete element.

| Measurements (mm) | AODF 0032 left pubis | AODF 0032 left ischium | AODF 0665 left pubis | AODF 0665 right pubis |
| --- | --- | --- | --- | --- |
| Proximodistal length | 940 | 379 | 948 | 732* |
| Proximal mediolateral width | 390 | 126 | 533* | 361 |
| Proximal dorsoventral breadth | 80 | 114 | 116 | 97 |
| Distal mediolateral width | 222 | 201 | 456 | 280 |
| Distal dorsoventral breadth | 36 | 25 | 94 | 83 |

**Table S14. Femur measurements**

**of AODF 0665 and AODF 0832.**

| Measurements (mm) | AODF 0665 right femur | AODF 0832 right femur |
| --- | --- | --- |
| Proximodistal length | 1505 | 1145 |
| Proximal end transverse width | 511 | 300 |
| Distance from proximal end to proximal end of fourth trochanter | 520 | 363 |
| Proximodistal length of fourth trochanter | 383 | 206 |
| Midshaft transverse width | 308 | 198 |
| Distal end maximum transverse width | 520 | 323 |

**Table S15. Astragalus measurements**

**of AODF 0666.**

| Measurements (mm) | right astragalus |
| --- | --- |
| Maximum transverse breadth | 198 |
| Maximum anteroposterior width | 131 |
| Maximum dorsoventral height | 129 |

**Table S16. Cervical vertebrae measurements**

**of AODF 0032.**

| Measurements (mm) | | A | B | C |
| --- | --- | --- | --- | --- |
| Centrum | Anteroposterior length (including condyle) | 675 | >650 | >250 |
|  | Anteroposterior length (excluding condyle) | 550 | - | - |
|  | Posterior cotyle dorsoventral height | - | ~183 | 219 |
|  | Posterior cotyle transverse width | - | ~270 | 311 |
| Pneumatic fossa | Anteroposterior length | - | - | 138 |
|  | Dorsoventral height | - | - | 63 |
| Transverse processes | Transverse width | - | - | >450 |

**Table S17. Humerus measurements**

**of AODF 0032.**

| Measurement (mm) | | Left humerus |
| --- | --- | --- |
| Proximodistal length | Proximal portion | 661 |
|  | Distal portion | 442 |
| Proximal mediolateral breadth | | 470 |
| Distal mediolateral breadth | | 450 |
