# Supplementary figures and images for: Reappraisal of sauropod dinosaur diversity in the Upper Cretaceous Winton Formation of Queensland, Australia, through 3D digitisation and description of new specimens
Source: PeerJ. 2024 Apr 9;12:e17180. doi: 10.7717/peerj.17180 (PMC11011616; doi:10.7717/peerj.17180)

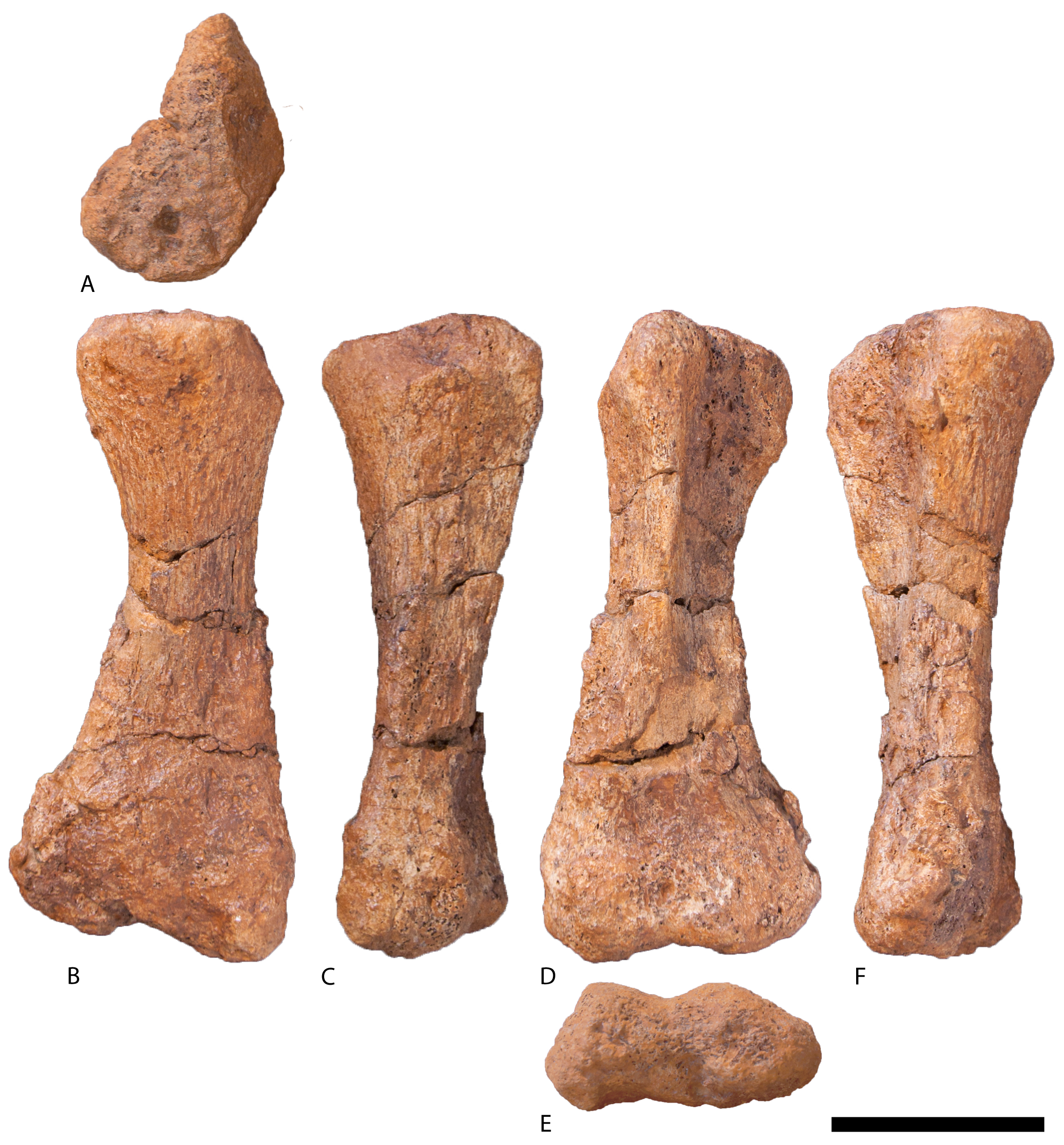

Supplement: Supplemental Information 5 — (A) proximal (B) anterior (C) medial (D) posterior (E) distal (F) lateral. Scale bar is 100 mm. [file peerj-12-17180-s005.png]

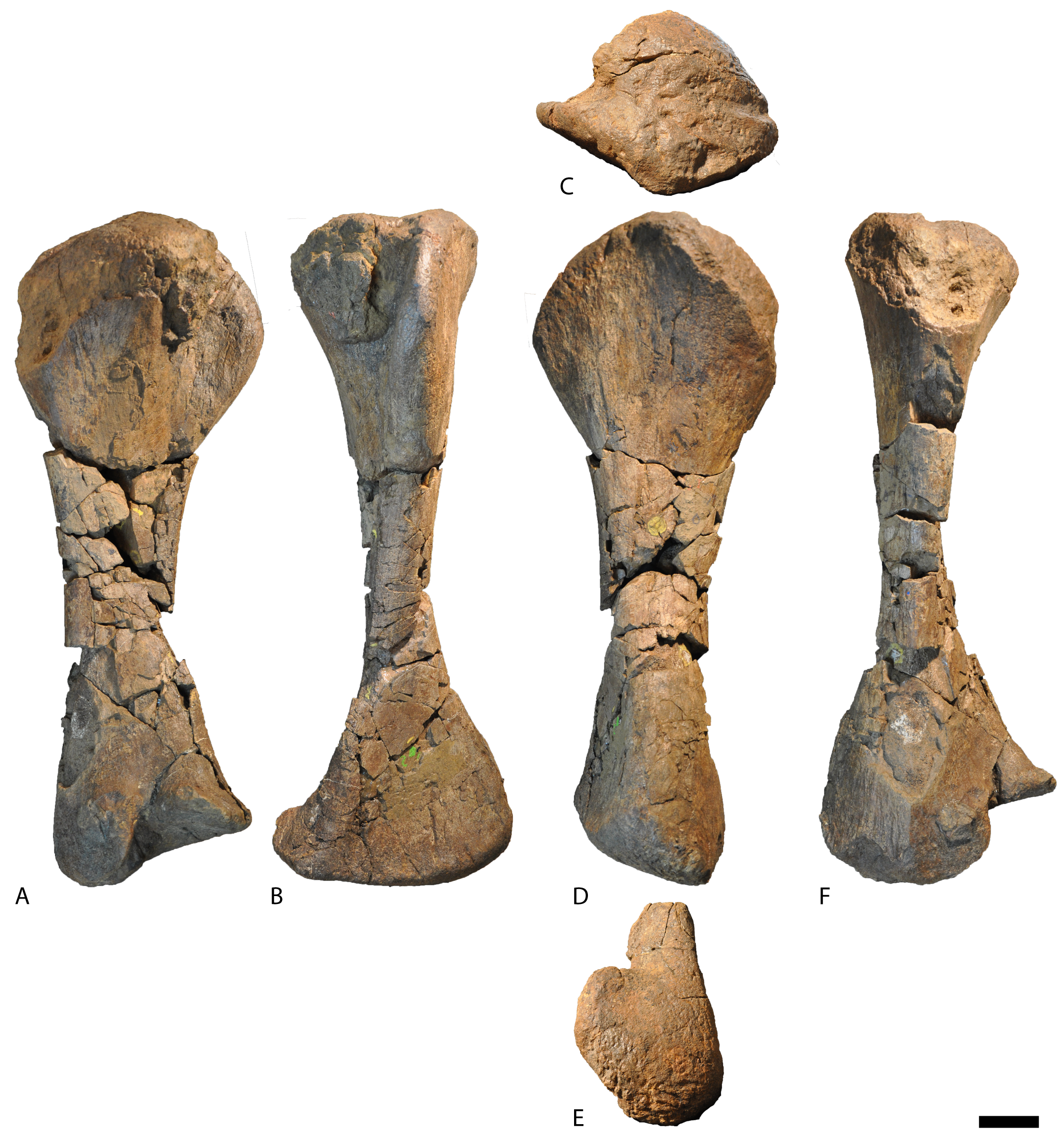

Supplement: Supplemental Information 6 — (A) lateral (B) anterior (C) proximal (D) medial (E) distal (F) posterior. Scale bar is 100 mm. [file peerj-12-17180-s006.png]

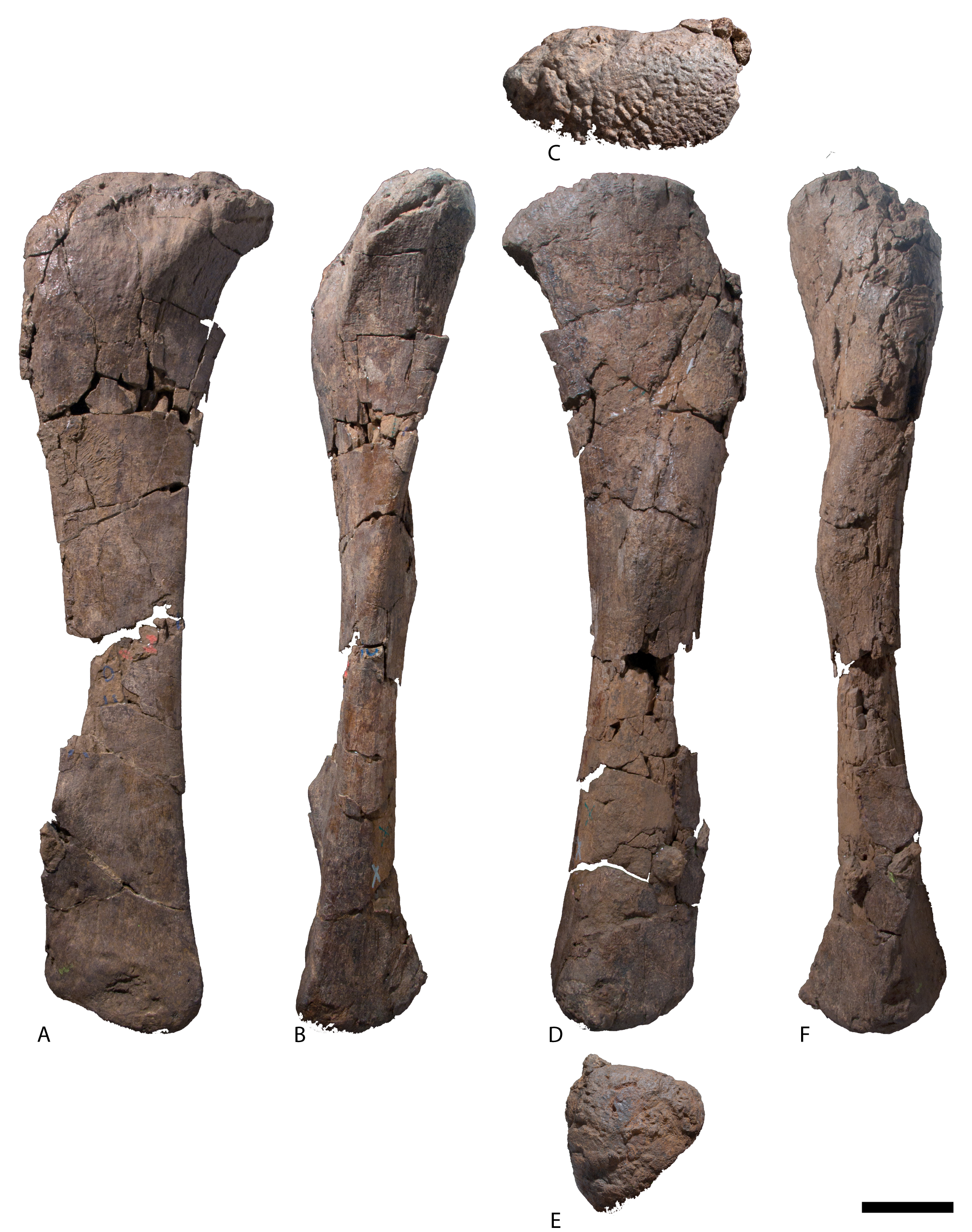

Supplement: Supplemental Information 7 — (A) medial (B) posterior (C) proximal (D) lateral (E) distal (F) anterior. Scale bar is 100 mm. [file peerj-12-17180-s007.png]

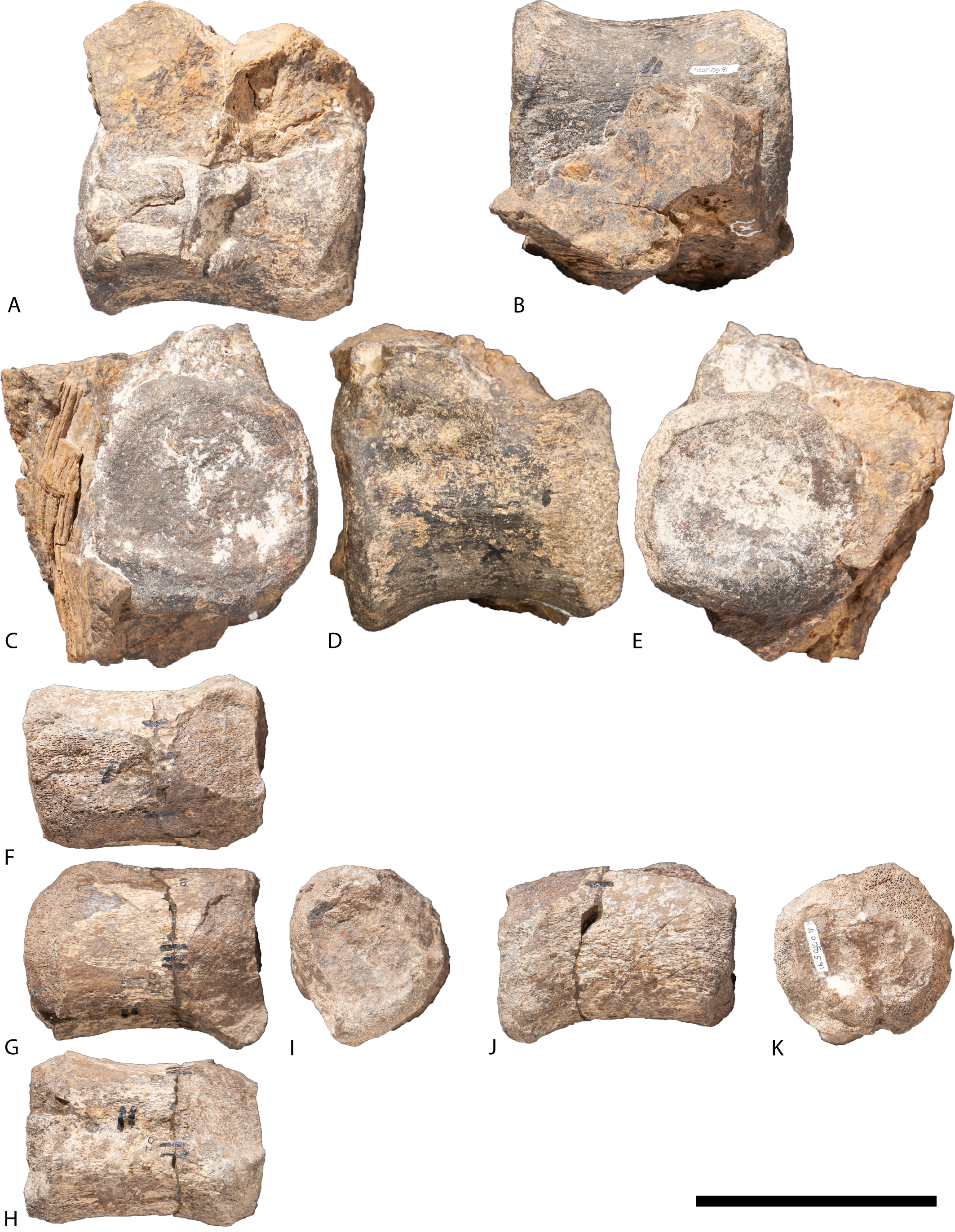

Supplement: Supplemental Information 8 — (A–E) Caudal vertebra A in (A) dorsal (B) ventral (C) anterior (D) left lateral (E) posterior. (F–K) Caudal vertebra B in (F) dorsal (G) left lateral (H) ventral (I) anterior (J) right lateral (K) posterior. Scale bar is 100 mm. [file peerj-12-17180-s008.png]

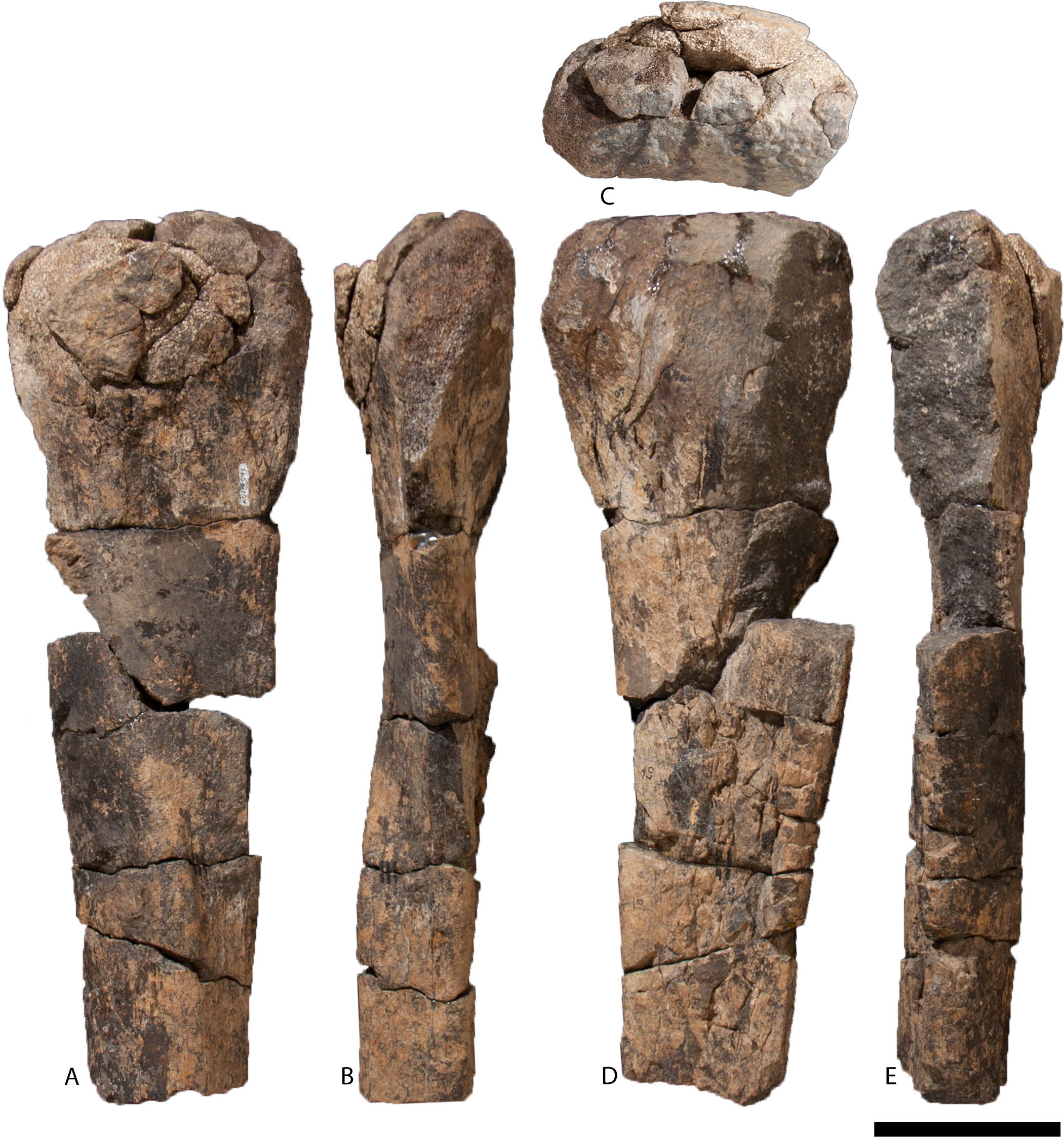

Supplement: Supplemental Information 9 — (A) lateral (B) posterior (C) proximal (D) medial (E) anterior. Scale bar is 100 mm. [file peerj-12-17180-s009.png]

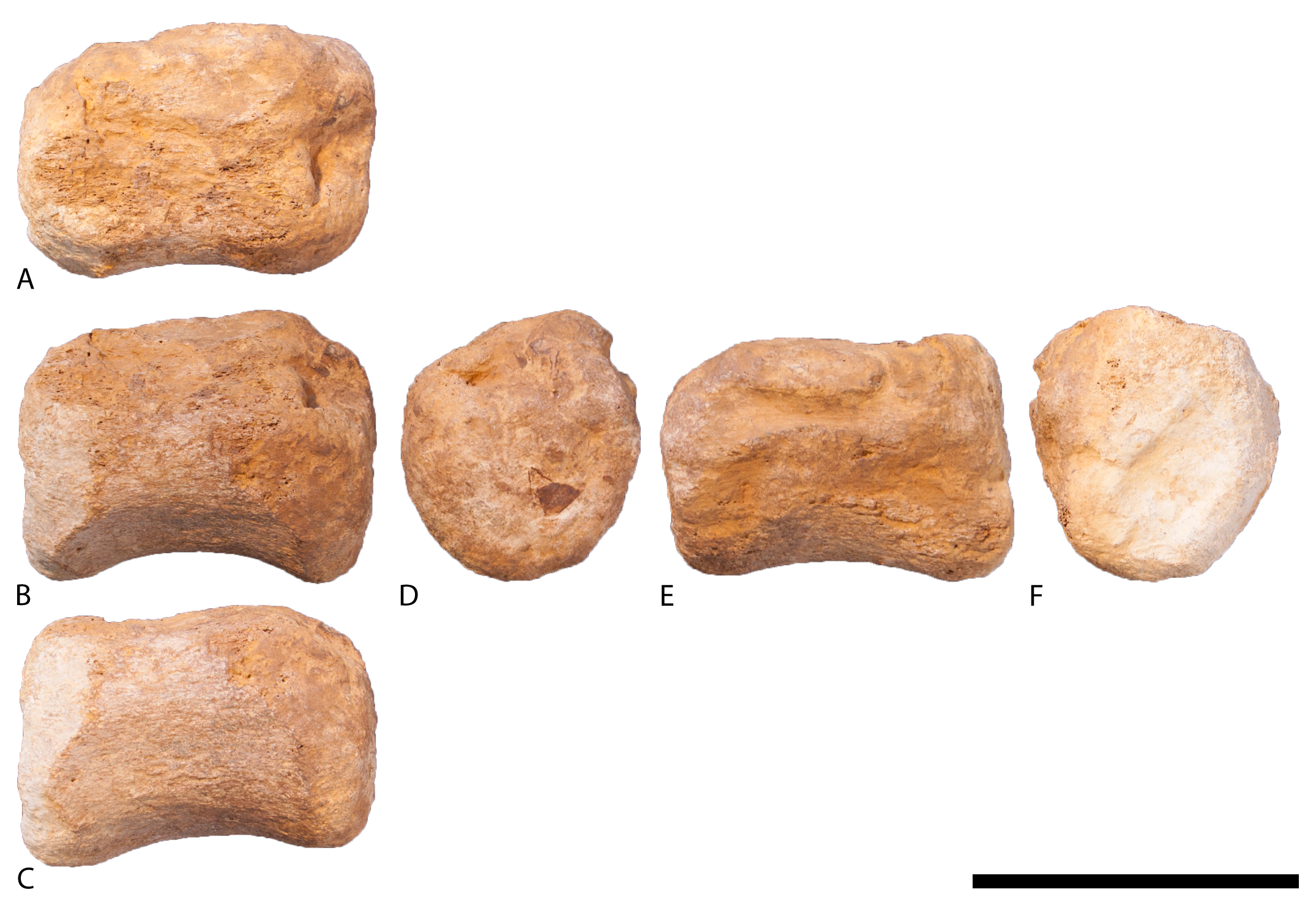

Supplement: Supplemental Information 10 — (A) right lateral (B) left lateral (C) ventral (D) anterior (E) dorsal (F) posterior. Scale bar is 100 mm. [file peerj-12-17180-s010.png]

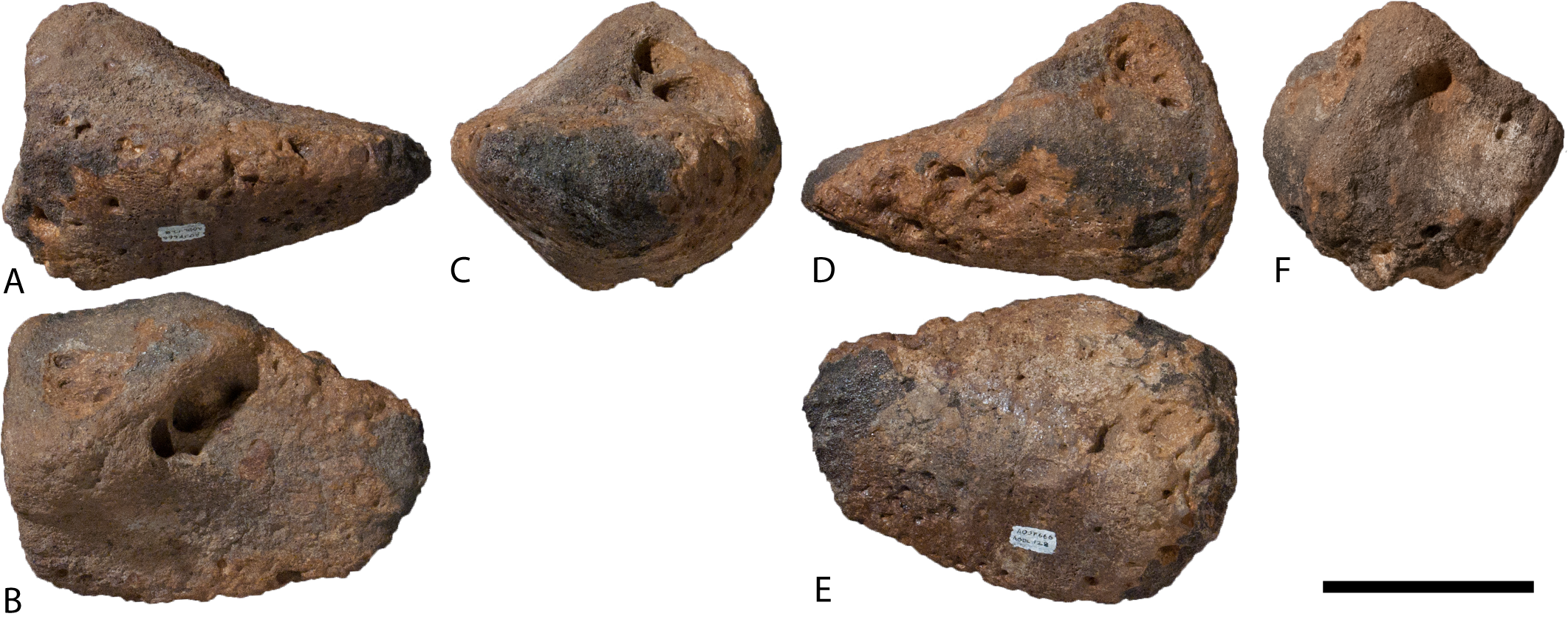

Supplement: Supplemental Information 11 — (A) anterior (B) proximal (C) medial (D) posterior (E) distal (F) lateral. Scale bar is 100 mm. [file peerj-12-17180-s011.png]

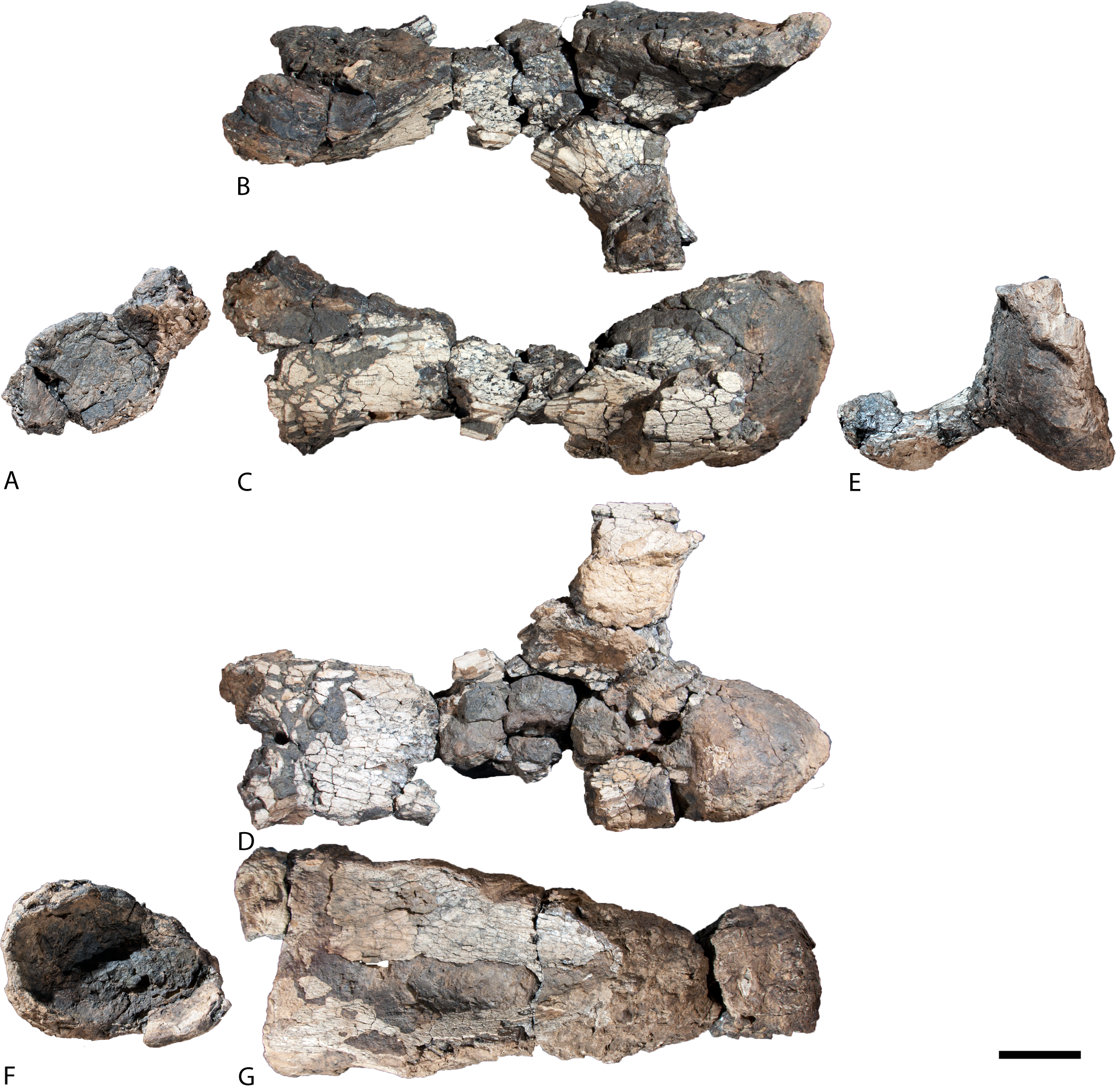

Supplement: Supplemental Information 12 — (A–D) cervical vertebra A in (A) posterior (B) dorsal (C) right lateral (D) ventral (E) anterior. (F–G) Cervical vertebra B in (F) posterior (G) ventral. Scale bar is 100 mm. [file peerj-12-17180-s012.png]

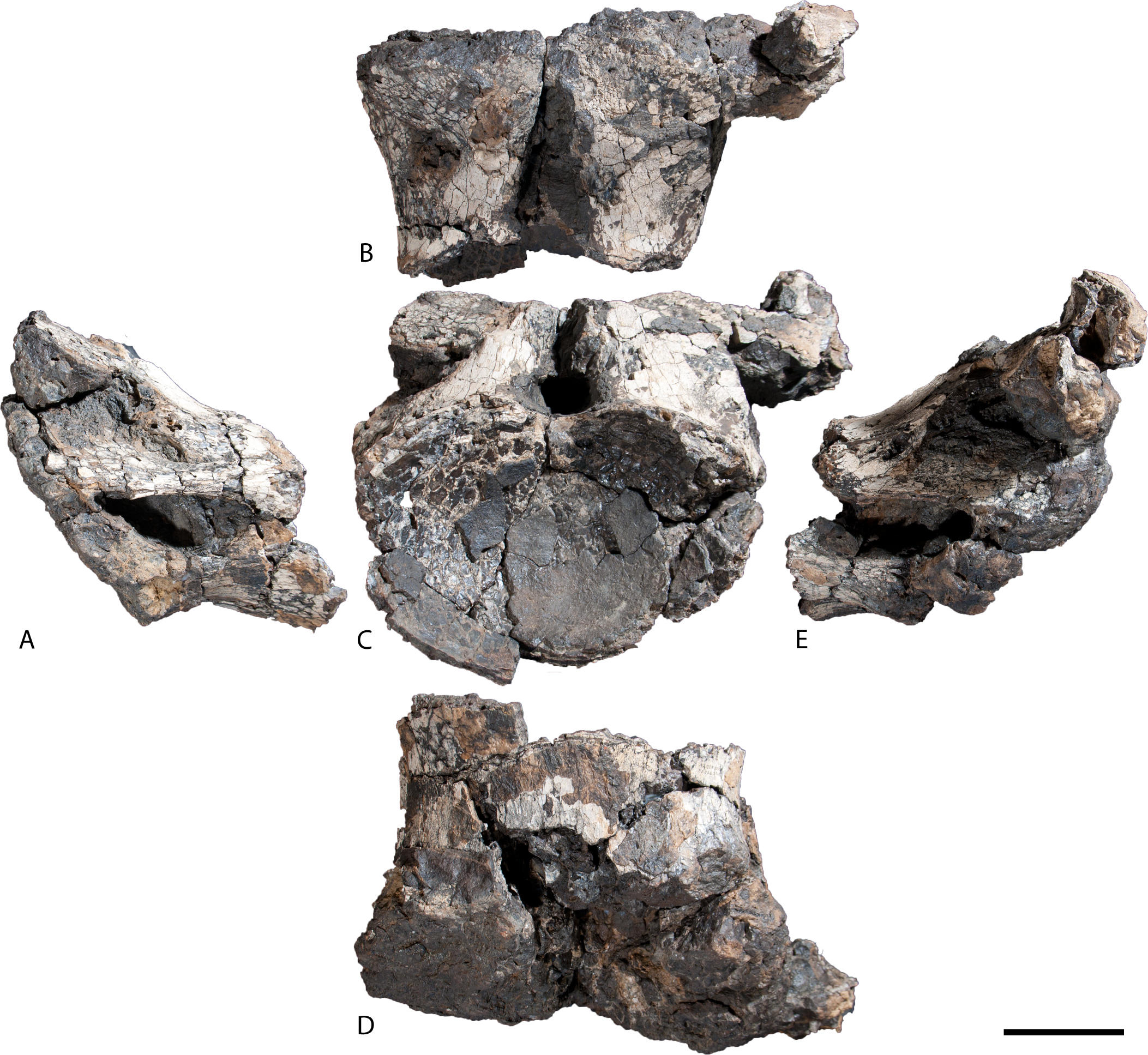

Supplement: Supplemental Information 13 — (A) left lateral (B) dorsal (C) posterior (D) ventral (E) right lateral. Scale bar is 100 mm. [file peerj-12-17180-s013.png]

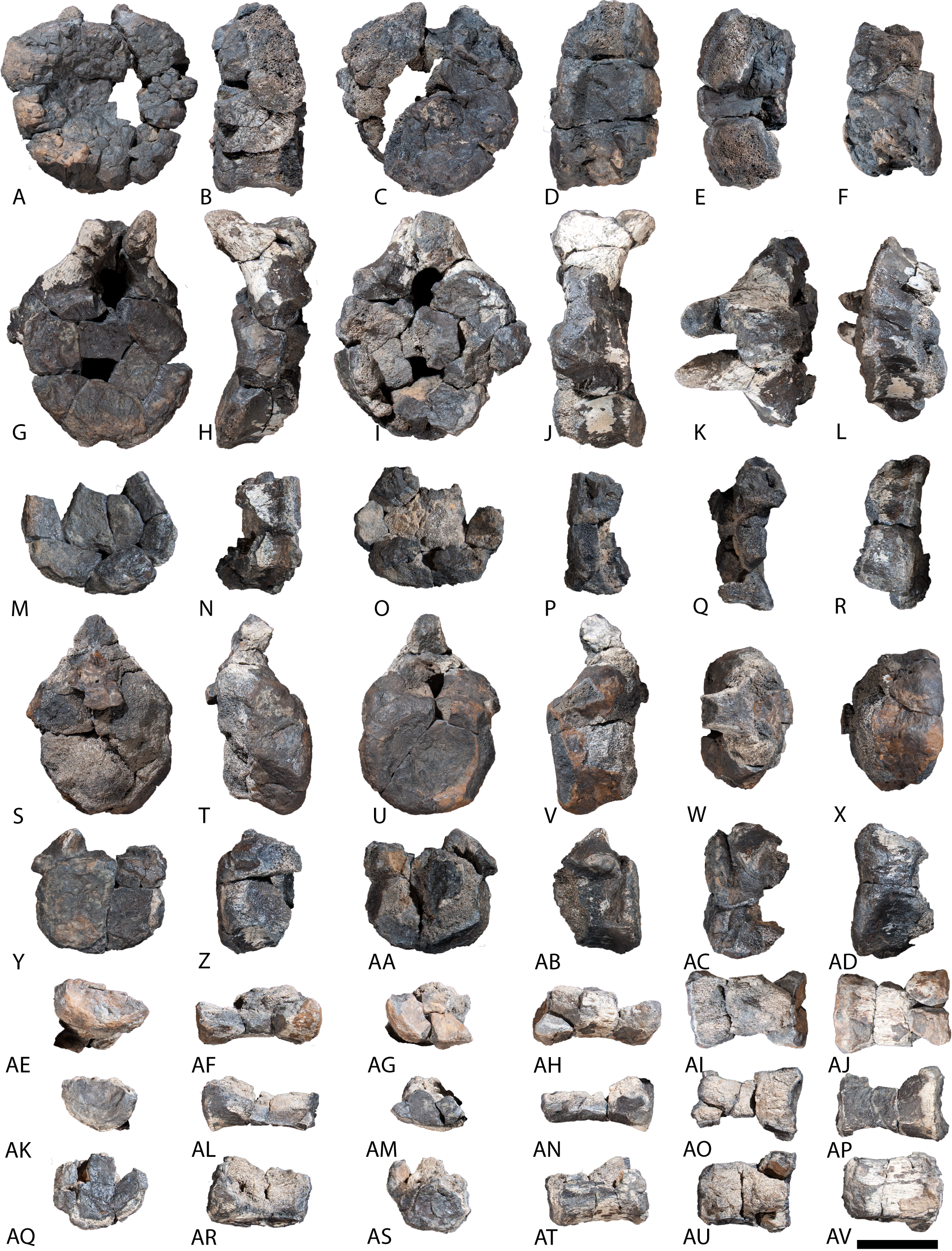

Supplement: Supplemental Information 14 — (A–F) Caudal vertebra A in (A) anterior (B) left lateral (C) posterior (D) right lateral (E) dorsal (F) ventral views. (G–L) Caudal vertebra B in (G) anterior (H) left lateral (I) posterior (J) right lateral (K) dorsal (L) ventral views. (M–R) Caudal vertebra C in (M) anterior (N) left lateral (O) posterior (P) right lateral (Q) dorsal (R) ventral views. (S–X) Caudal vertebra D in (S) anterior (T) left lateral (U) posterior (V) right lateral (W) dorsal (X) ventral views. (Y–AD) Caudal vertebra E in (Y) anterior (Z) left lateral, (AA) posterior (AB) right lateral (AC) dorsal (AD) ventral views. (AE–AJ) Caudal vertebra F in (AE) anterior (AF) left lateral, (AG) posterior (AH) right lateral (AI) dorsal (AJ) ventral views. (AK–AP) Caudal vertebra G in (AK) anterior (AL) left lateral, (AM) posterior (AN) right lateral (AO) dorsal (AP) ventral views. (AQ–AV) Caudal vertebra H in (AQ) anterior (AR) left lateral, (AS) posterior (AT) right lateral (AU) dorsal (AV) ventral views. Scale bar is 100 mm. [file peerj-12-17180-s014.png]

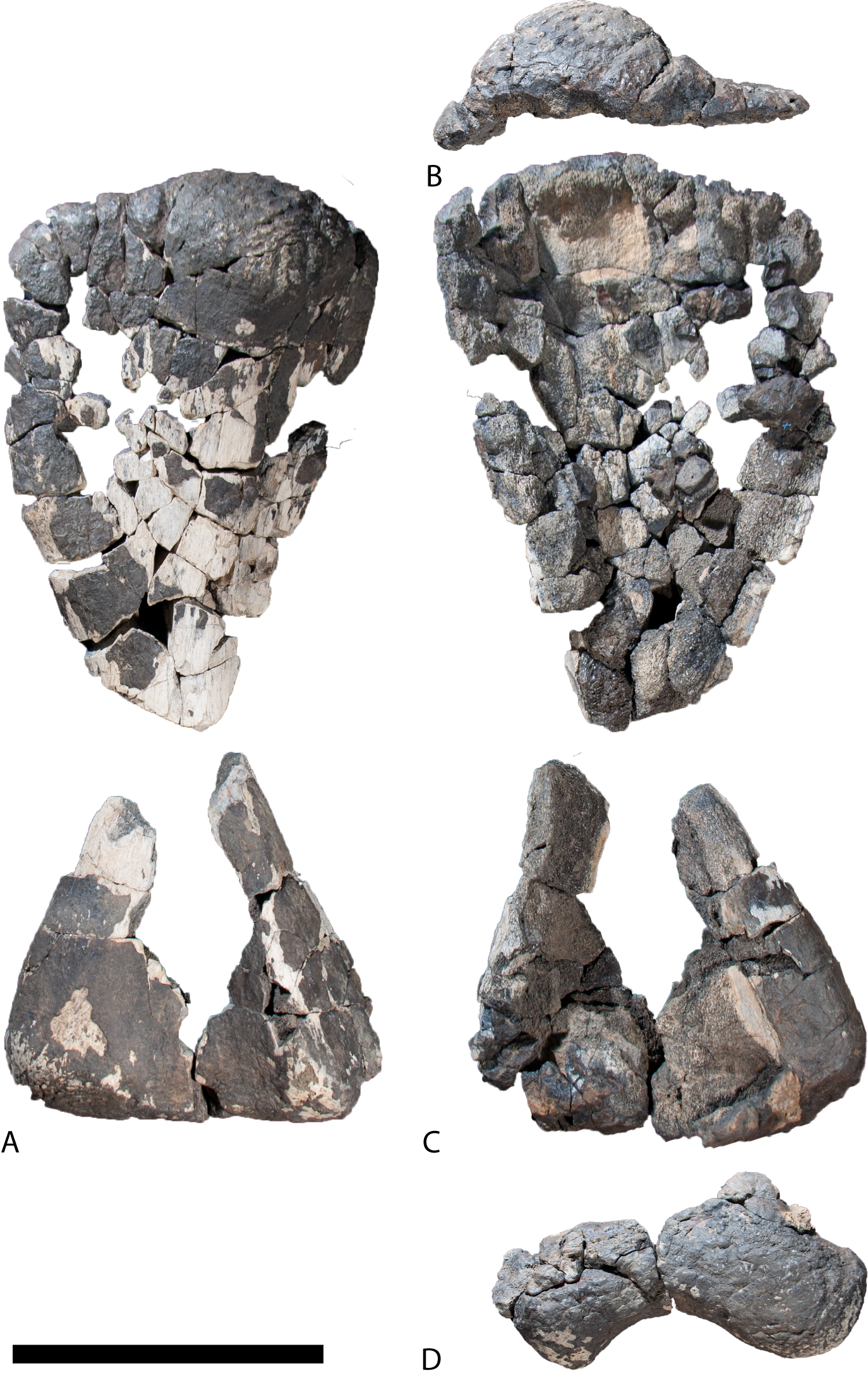

Supplement: Supplemental Information 15 — (A) posterior (B) proximal (C) anterior (D) distal. Scale bar is 500 mm. [file peerj-12-17180-s015.png]

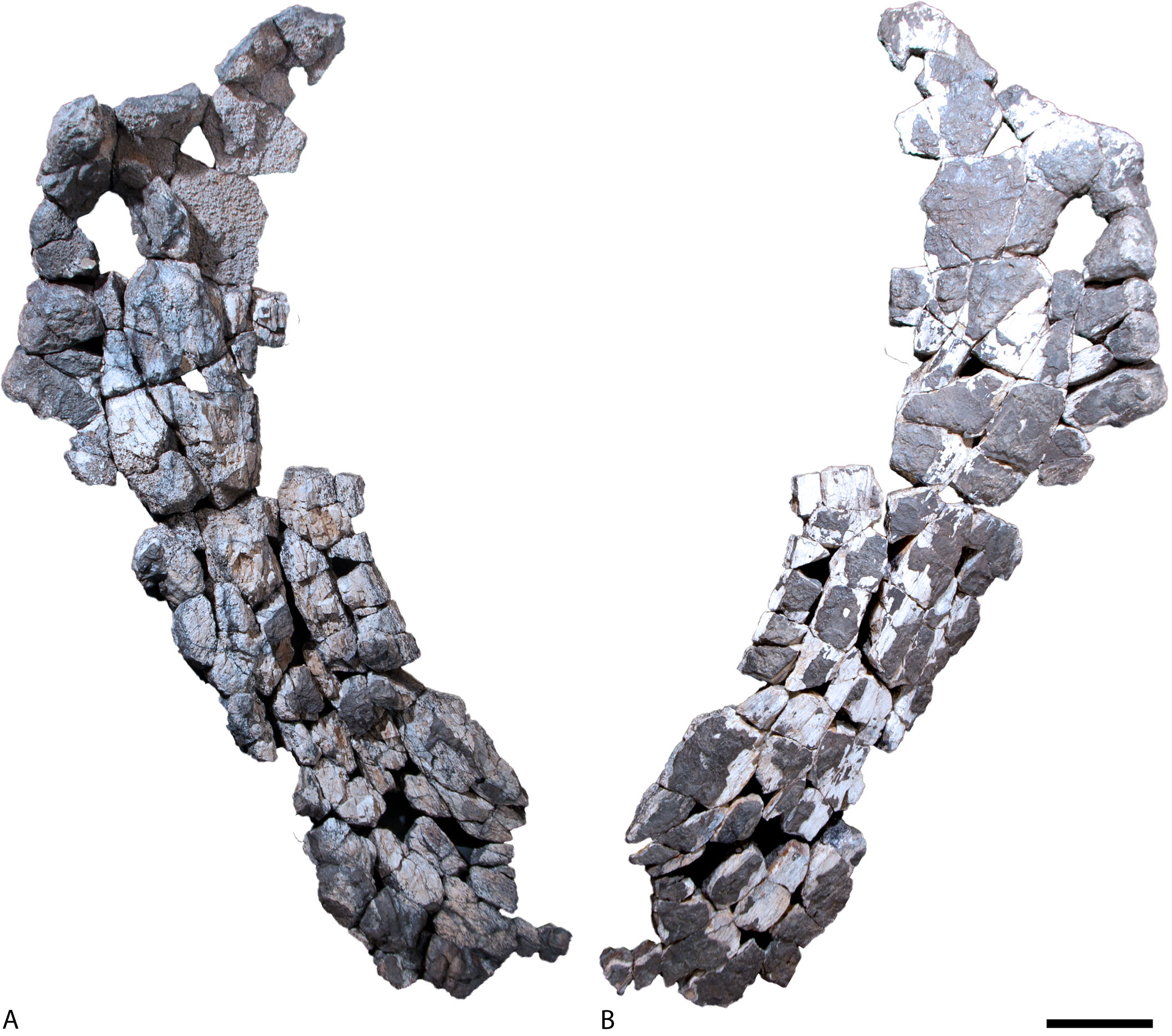

Supplement: Supplemental Information 16 — (A) medial (B) lateral. Scale bar is 100 mm. [file peerj-12-17180-s016.png]

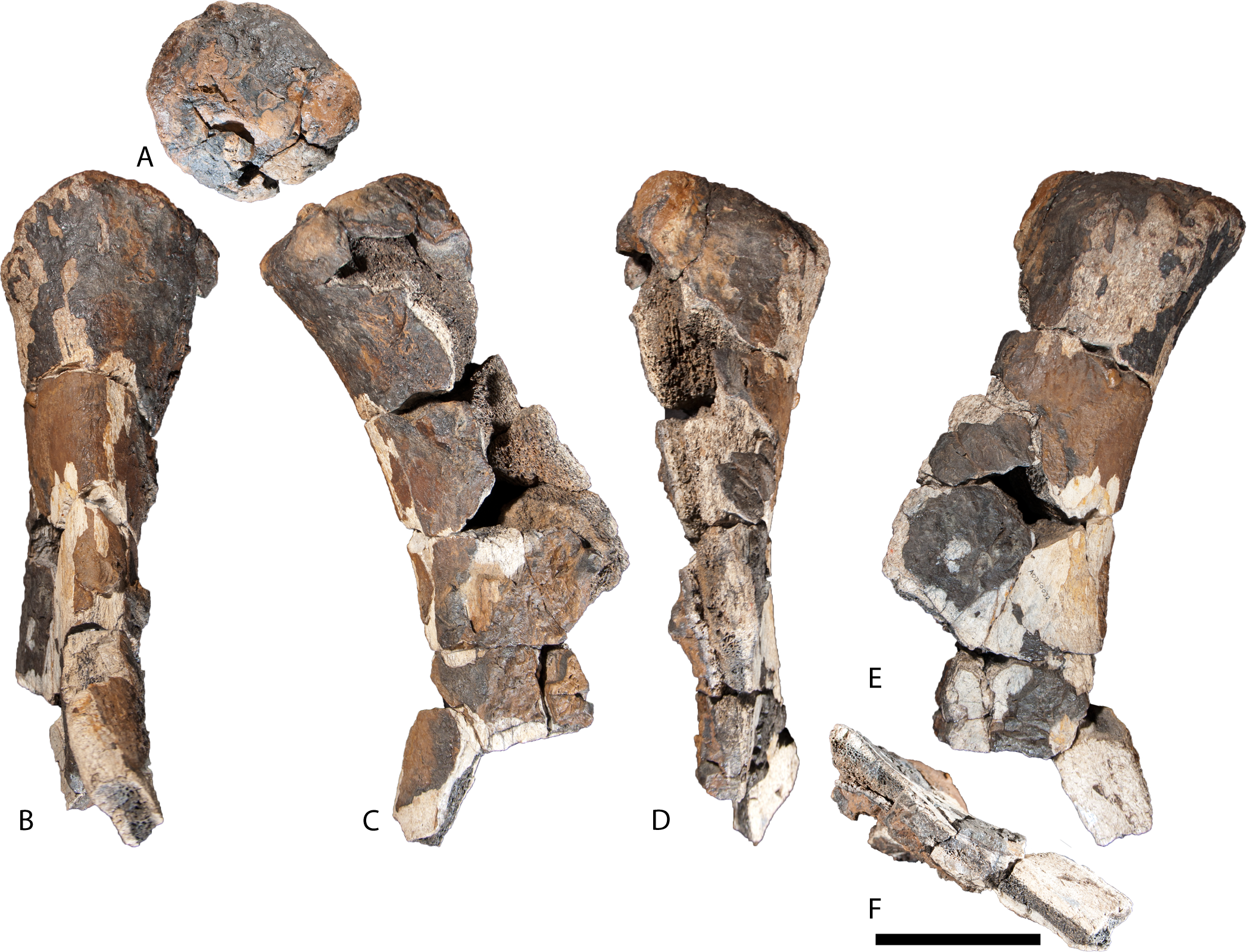

Supplement: Supplemental Information 17 — (A) proximal (B) posterior (C) medial (D) anterior (E) lateral (F) distal. Scale bar is 100 mm. [file peerj-12-17180-s017.png]

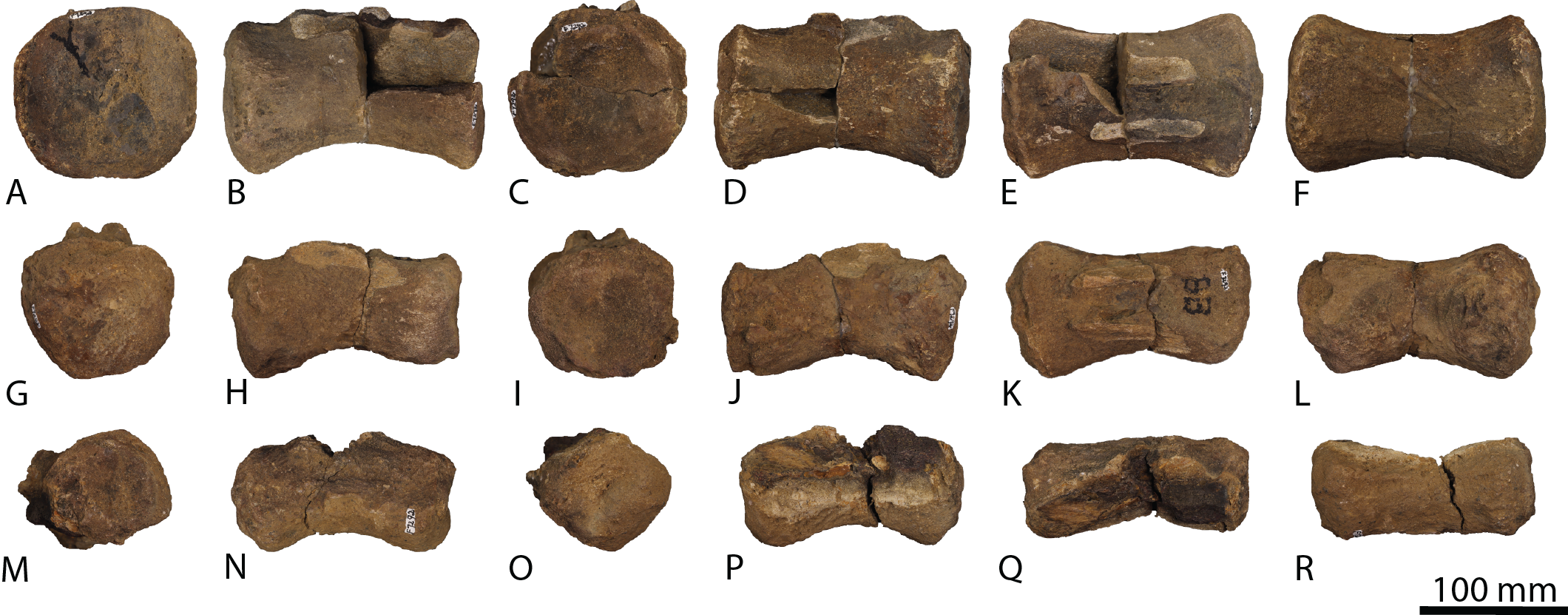

Supplement: Supplemental Information 18 — (A–F) Additional caudal vertebra 1 in (A) anterior (B) left lateral (C) posterior (D) right lateral (E) dorsal (F) ventral views. (G–L) Additional caudal vertebra 2 in (G) anterior (H) left lateral (I) posterior (J) right lateral (K) dorsal (L) ventral views. (M–R) Additional caudal vertebra 3 in (M) anterior (N) left lateral (O) posterior (P) right lateral (Q) dorsal (R) ventral views. [file peerj-12-17180-s018.png]
